# Mediterranean diet adherence and rate of cerebral Aβ-amyloid accumulation: Data from the Australian Imaging, Biomarkers and Lifestyle Study of Ageing

**DOI:** 10.1038/s41398-018-0293-5

**Published:** 2018-10-30

**Authors:** Stephanie R. Rainey-Smith, Yian Gu, Samantha L. Gardener, James D. Doecke, Victor L. Villemagne, Belinda M. Brown, Kevin Taddei, Simon M. Laws, Hamid R. Sohrabi, Michael Weinborn, David Ames, Christopher Fowler, S. Lance Macaulay, Paul Maruff, Colin L. Masters, Olivier Salvado, Christopher C. Rowe, Nikolaos Scarmeas, Ralph N. Martins

**Affiliations:** 1 Centre of Excellence for Alzheimer’s disease Research and Care, School of Medical and Health Sciences, Edith Cowan University, Joondalup, Western Australia Australia; 2 Sir James McCusker Alzheimer’s Disease Research Unit (Hollywood Private Hospital), Perth, Western Australia Australia; 3 Taub Institute for Research of Alzheimer’s Disease and the Ageing Brain, Columbia University, New York, NY USA; 4 Gertrude H. Sergievsky Centre, Columbia University, New York, NY USA; 5 CSIRO Health and Biosecurity/Australian e-Health Research Centre, Brisbane, Queensland Australia; 6 Cooperative Research Centre for Mental Health, Carlton, VIC Australia; 7 Department of Nuclear Medicine and Centre for PET, Austin Health, Heidelberg, Victoria Australia; 8 School of Psychology and Exercise Science, Murdoch University, Murdoch, Western Australia Australia; 9 Collaborative Genomics Group, Centre of Excellence for Alzheimer’s disease Research and Care, School of Medical and Health Sciences, Edith Cowan University, Joondalup, Western Australia Australia; 10 School of Biomedical Sciences, Faculty of Health Sciences, Curtin Health Innovation Research Institute, Curtin University, Bentley, Western Australia Australia; 11 School of Biomedical Sciences, Macquarie University, Macquarie Park, New South Wales Australia; 12 School of Psychological Science, University of Western Australia, Crawley, Western Australia Australia; 13 National Ageing Research Institute, Parkville, Victoria Australia; 14 Academic Unit for Psychiatry of Old Age, St. Vincent’s Health, Department of Psychiatry, The University of Melbourne, Kew, Victoria Australia; 15 The Florey Institute of Neuroscience and Mental Health, The University of Melbourne, Parkville, Victoria Australia; 16 CogState Ltd., Melbourne, Victoria, Australia; 17 Department of Neurology, Columbia University College of Physicians and Surgeons, New York, NY USA; 18 Department of Social Medicine, Psychiatry, and Neurology, National and Kapodistrian University of Athens, Athens, Greece; 19 School of Psychiatry and Clinical Neurosciences, University of Western Australia, Nedlands, Western Australia Australia

## Abstract

Accumulating research has linked Mediterranean diet (MeDi) adherence with slower cognitive decline and reduced Alzheimer’s disease (AD) risk. However, no study to-date has examined the relationship between MeDi adherence and accumulation of cerebral Aβ-amyloid (Aβ; a pathological hallmark of AD) in older adults. Cognitively normal healthy control participants of the Australian Imaging, Biomarkers and Lifestyle (AIBL) Study of Ageing completed the Cancer Council of Victoria Food Frequency Questionnaire at baseline, which was used to construct a MeDi score for each participant (score range 0–9; higher score indicating higher adherence). Cerebral Aβ load was quantified by Pittsburgh Compound B positron emission tomography at baseline, 18 and 36 months: Only individuals categorised as “Aβ accumulators”, and thus considered to be on the AD pathway, were included in the analysis (*N* = 77). The relationship between MeDi adherence, MeDi components, and change in cerebral Aβ load (baseline to 36 months) was evaluated using Generalised Linear Modelling, accounting for age, gender, education, Apolipoprotein E ε4 allele status, body mass index and total energy intake. Higher MeDi score was associated with less Aβ accumulation in our cohort (β = −0.01 ± 0.004, *p* = 0.0070). Of the individual MeDi score components, a high intake of fruit was associated with less accumulation of Aβ (β = −0.04 ± 0.01, *p* = 0.00036). Our results suggest MeDi adherence is associated with reduced cerebral AD pathology accumulation over time. When our results are considered collectively with previous data linking the MeDi to slower cognitive decline, it is apparent that MeDi adherence warrants further investigation in the quest to delay AD onset.

## Introduction

Currently, no cure for Alzheimer’s disease (AD) exists, it is therefore essential that effective and broadly applicable approaches to prevent or delay disease onset are developed. Accumulating data suggests that diet represents one such strategy accessible to all.

The Mediterranean diet (MeDi), has been widely recognised as a healthy eating model due to its correlation with low morbidity and mortality for many chronic diseases, including cardiovascular disease and diabetes^1,2^, which are themselves risk factors for AD^3,4^. Indeed, accumulating research, including our own, has linked MeDi adherence with slower cognitive decline and reduced risk of AD^5–7^. Precisely how the MeDi confers such cognitive benefit remains to be determined; brain imaging studies likely offer some insight. Relatively few studies however, have examined the relationship of MeDi adherence to AD-related neuroimaging outcome measures. Higher adherence to MeDi has been associated with greater cortical thickness^8^, whilst similarly, lower MeDi adherence has been shown to be predictive of increased total brain atrophy^9^. To-date, only one study has directly examined the relationship between MeDi adherence and cerebral Aβ-amyloid (Aβ) load (a pathological hallmark of AD), with the authors reporting reduced cerebral Aβ load cross-sectionally among individuals with high MeDi adherence^10^.

There is a critical need to explore the relationship between MeDi and rates of cerebral Aβ accumulation using longitudinal data collected from a well-characterised ageing cohort. Consequently, the aim of the current study was to assess the relationship between MeDi adherence, MeDi components, and Pittsburgh Compound B positron emission tomography (PiB-PET) determined cerebral Aβ burden longitudinally, in cognitively normal healthy control participants of the Australian Imaging, Biomarkers and Lifestyle (AIBL) Study of Ageing, who were categorised as “Aβ accumulators”, and thus considered to be on the AD pathway. We hypothesised that higher MeDi adherence would be associated with less accumulation of cerebral Aβ over time.

## Methods

### Participants

This report describes data from 77 participants taken from the AIBL Study of Ageing^11^ who were classified as healthy controls (cognitively ‘normal’) and completed the Cancer Council of Victoria Food Frequency Questionnaire (CCVFFQ) at baseline, and who were categorised as “Aβ accumulators”^12^ as determined by PiB-PET imaging undertaken to assess cerebral Aβ levels at baseline, 18 and 36-month follow-up assessments. Baseline assessments commenced in October 2006, and 36-month follow-up assessments were completed in October 2011. All volunteers were aged 60 years and above at baseline, and further details regarding recruitment, assessment, inclusion, and exclusion criteria are described in Ellis et al^11^. The AIBL Study is approved by the institutional ethics committees of Austin Health, St Vincent’s Health, Hollywood Private Hospital and Edith Cowan University^11^. Written informed consent was obtained from each participant prior to undertaking any procedures related to the study.

### Food frequency questionnaires

The CCVFFQ is a 74 single item, semi-quantitative, machine-scannable FFQ, which is optically scanned to provide grams per day of food and nutrient intake. The CCVFFQ has been validated relative to seven-day weighed food records in pre-menopausal women^13,14^. The completed questionnaires are analysed by the Cancer Council in Carlton, Victoria. The food composition data used to calculate daily nutrient intake originates from the Nutrient Tables for use in Australia 1995 NUTTAB95;^15^ the CCVFFQ takes approximately ten minutes to complete and assesses usual daily intake over the preceding 12 months. Participants completed the CCVFFQ when they attended the research centre for assessment.

The CCVFFQ data were used to construct a MeDi score for each participant using the most commonly described method;^16^ however, cohort sex-specific medians rather than population sex-specific medians were used. A value of 1 was assigned for; (i) each beneficial component (fruits, vegetables, legumes, cereals and fish) if caloric-adjusted consumption was at or above the cohort sex-specific median; (ii) each detrimental component (meat and dairy) if caloric-adjusted consumption was below the sex-specific median; and (iii) a ratio of monounsaturated to saturated fats at or above the median. Individuals were assigned a value of 1 for mild-to-moderate alcohol consumption (>5 to <25 g per day for females and >10 to <50 g per day for males). A MeDi score was subsequently generated for each participant by summing the scores for each category; MeDi score ranged from 0–9, with higher score indicating greater adherence^5^.

### Aβ-amyloid imaging

Cerebral Aβ load was assessed at baseline, 18 and 36-month follow-up using 11C-Pittsburgh Compound B (PiB) positron emission tomography (PET). PET methodology has previously been described in detail^12^. Briefly, a 30-minute acquisition was performed 40 min post-injection of PiB. For semi-quantitative analysis, a volume of interest template was applied to the summed and spatially normalised PET images in order to calculate a standardised uptake value (SUV). Images were then scaled to the SUV of the cerebellar cortex (reference region) to generate a tissue ratio termed SUV ratio (SUVR). Global SUVR was calculated using the mean SUVR in the frontal, superior parietal, lateral temporal, occipital and anterior and posterior cingulate regions. Only individuals categorised as “Aβ accumulators” were included in the current study; i.e., participants with an already high Aβ burden (SUVR ≥1·4) in the brain at baseline, or those who, despite having a low Aβ burden (SUVR <1·4) in the brain, presented with rates of Aβ accumulation higher than 0 over 36 months^12^.

### Apolipoprotein E genotyping

Fasting blood samples were obtained using standard venepuncture of the antecubital vein and collected into EDTA tubes containing Prostaglandin E1 (PGE; Sapphire Bioscience, NSW, Australia, 33·3 ng/ml) to prevent platelet activation. Extraction of DNA from 5 ml of whole blood was undertaken using QIAamp DNA Blood Maxi Kits (Qiagen, Hilden, Germany) as per the manufacturer’s instructions. Specific TaqMan® (Thermo Fisher Scientific, Waltham, MA, USA) genotyping assays were used for ascertaining Apolipoprotein E (*APOE*) genotype (rs7412, assay ID: C____904973_10; rs429358, assay ID: C___3084793_20), which were performed on a QuantStudio 12 K Flex™ Real-Time-PCR system (Thermo Fisher Scientific, Waltham, MA, USA) using the TaqMan® GTXpress™ Master Mix (Thermo Fisher Scientific, Waltham, MA, USA).

### Statistical analysis

Means, standard deviations and percentages are provided for the demographics of the entire cohort (Table 1). Demographic group differences were evaluated using independent sample *t*-tests for continuous variables and chi-square (χ^2^) analysis for categorical variables. MeDi score components were transformed initially to remove those outliers that were obviously erroneous data, with the erroneous data replaced with median values.

**Table 1:** Descriptive statistics for the cognitively normal healthy control “Aβ accumulator” cohort who completed the CCVFFQ at baseline, and underwent PiB-PET imaging to assess cerebral Aβ levels at baseline, 18 and 36-month follow-up assessments

	Total sample (*N* = 77)
Gender male, *N* (%)	39 (51)
Mean age, years (SD)	71.1 (7.1)
*APOE* ε4 carriage, *N* (%)	32 (42)
Mean BMI at baseline (SD)^a^	26.2 (3.2)
Years of education >12 years, *N* (%)	44 (57)
Mean MMSE at baseline (SD)	29 (2)
Median MeDi score (IQR)	4 (2)
SUVR < 1.4 at baseline, *N* (%)	50 (65)

*Aβ* Aβ-amyloid; *APOE* apolipoprotein E, *BMI* body mass index, *CCVFFQ* Cancer Council of Victoria Food Frequency Questionnaire, *IQR* interquartile range, *MeDi* Mediterranean diet, *MMSE* Mini-Mental State Examination, *PiB-PET* 11C-Pittsburgh Compound B positron emission tomography, *SD* standard deviation, *SUVR* standardised uptake value ratio

^a^Body mass index is calculated as weight in kilograms divided by height in meters squared

Change in Aβ over 36 months was represented using the beta coefficients from a robust linear model, whereby individual SUVR values were modelled against time (baseline, 18 and 36 months). Two separate modelling (Generalised Linear Model, GLM) strategies considered; (1) assessment of the total MeDi score alone (adjusted for covariates, with and without *APOE* ε4 allele status interaction), and (2) assessment of all the individual components of the total MeDi score (intake of meat, fish, vegetables, cereals, legumes, dairy, fruit, alcohol per day and the ratio of monounsaturated fatty acids to saturated fatty acids; including covariates; excluding total MeDi score) using a stepwise linear model. The stepAIC (Akaike information criterion) function was used to identify the best model, before reducing to the strongest components. Covariate factors included age, gender, education (2 groups; ≤12 years/>12 years), *APOE* ε4 allele status (2 groups; absence of ε4 allele/ presence of either one or two ε4 alleles) and body mass index (BMI). From the final models, *p-*values less than 0.05 were classed statistically significant. All statistical analyses were performed using R version 3.3.2 (2016-10-31; R Foundation for Statistical Computing, Vienna, Austria). Visualisation of the MeDi score with change in SUVR was investigated using traditional tertile transformation.

## Results

The cohort comprised 77 cognitively normal healthy control “Aβ accumulator” participants (51% male) with an average age of 71.1 ± 7.1 years. Forty-two per cent were carriers of at least one *APOE* ε4 allele (the most common genetic risk factor for AD) and more than half had 13 or more years of education. The median MeDi score of the cohort was 4 (interquartile range 2) and 65% of participants had an SUVR less than 1.4 at baseline (Table 1).

When assessing the effect of MeDi score on the change in Aβ over 36 months, adjusting for age, gender and *APOE* ε4 allele status, an inverse relationship was seen, with increasing MeDi score associated with decreasing SUVR (β = −0.01 ± 0.004, *p* = 0.0070). Given the average accumulation rate of Aβ in this cohort was 0.05 SUVR units/year, altering the diet of an individual to increase MeDi score by one point would result in an approximate 20% decrease in Aβ accumulation over a one year period, and up to a 60% decrease in accumulation over three years.

When evaluating which individual components of the total MeDi score were contributing most strongly to the decreased rate of Aβ accumulation, the optimal model from the stepwise regression (based on the AIC; including all covariates and MeDi score components; excluding total MeDi score) included age (β = 0.002 ± 0.0008, *p* = 0.0083), *APOE* ε4 allele status (β = 0.03 ± 0.01, *p* = 0.0095), and the intakes of fruit (β = -0.04 ± 0.01, *p* = 0.00085), meat (β = −0.02 ± 0.01, *p* = 0.057), cereals (β = −0.02 ± 0.01, *p* = 0.13) and dairy (β = 0.02 ± 0.01, *p* = 0.13). Reducing this to a minimalistic model given the sample size, the final model included age (β = 0.002 ± 0.0008, *p* = 0.020), *APOE* ε4 allele status (β = 0.03 ± 0.01, *p* = 0.021) and fruit intake (β = −0.04 ± 0.01, *p* = 0.00036). Covariates not included in the final model did not contribute towards variance in the outcome, and were removed via the stepAIC procedure. Complete results from the GLM are presented in Table 2.

**Table 2:** GLM coefficients: MeDi score to predict change in SUVR over 36 months

*Model* / Variable	β coefficient	SE	*t*-value	*p*-value
*Initial model including total MeDi score (without MeDi score components):*				
(Intercept)	−0.02905	0.06602	−0.44006	0.66120
MeDi Score	−0.01015	0.00366	−2.77308	**0.00704**
*APOE* ε4 allele status	0.03134	0.01228	2.55229	**0.01279**
Age	0.00164	0.00086	1.90979	0.06009
*Second model including MeDi score components (without total MeDi score):*				
(Intercept)	−0.08644	0.06313	−1.36934	0.17527
Fruit intake (0/1)	−0.03802	0.01091	−3.48617	**0.00085**
Age	0.00225	0.00083	2.71501	**0.00834**
*APOE* ε4 allele status	0.03061	0.01147	2.66877	**0.00945**
Meat intake (0/1)	−0.02215	0.01144	−1.93659	0.05683
Cereals intake (0/1)	−0.01758	0.01150	−1.52890	0.13080
Dairy intake (0/1)	0.01705	0.01118	1.52563	0.13161
*Second model (reduced)*				
(Intercept)	−0.07342	0.06033	−1.217	0.22760
Fruit intake (0/1)	−0.04143	0.01107	−3.744	**0.00036**
Age	0.00196	0.00082	2.385	**0.01967**
*APOE* ε4 allele status	0.02749	0.01170	2.350	**0.02148**
*Interaction model (Total MeDi score)*				
(Intercept)	−0.03027	0.06612	−0.458	0.64850
MeDi score	−0.01245	0.00447	−2.786	**0.00681**
Age	0.00178	0.00087	2.037	**0.04532**
*APOE* ε4 allele status	0.00157	0.03526	0.045	0.96453
MeDi score * *APOE* ε4 allele status	0.00701	0.00780	0.901	0.37061

All beta (β) coefficients (± SE) from the GLM are shown. Bold indicates statistical significance (*p* < 0.05)

*APOE* apolipoprotein E, *GLM* generalised linear model, *MeDi* Mediterranean diet, *SE* standard error, *SUVR* standardised uptake value ratio

No significant interaction between MeDi score and *APOE* ε4 allele status was observed for the change in SUVR via the GLM (β = 0.007 ± 0.008, *p* = 0.37). The beta coefficient and resultant *p*-value for MeDi score however were moderately stronger (β = −0.012 ± 0.004, *p* = 0.0068), while the beta coefficient and resultant *p*-value for *APOE* ε4 allele status was abrogated (β = 0.002 ± 0.04, *p* = 0.96; Table 2).

**Fig. 1:**
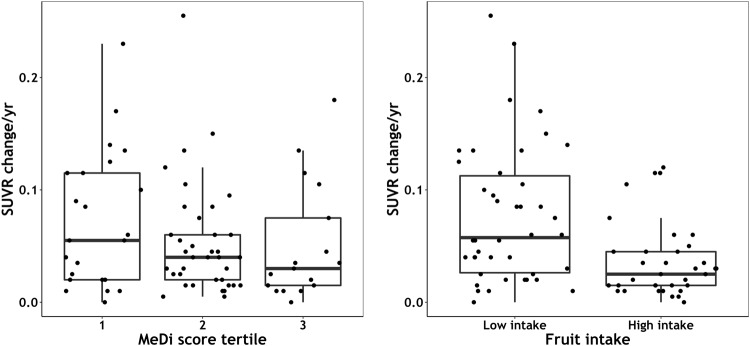
Plots demonstrate the relationship between MeDi score in tertiles and median change in SUVR per year (left), and fruit intake and median change in SUVR per year (right). MeDi score tertile 1 = lowest MeDi adherence; 3 = highest MeDi adherence. Fruit intake was categorised as ‘low’ or ‘high’ using cohort sex-specific caloric-adjusted medians as the cut-off. Upper horizontal line shows the third quartile above the median change in SUVR per year, middle horizontal line is the median change, and lower horizontal line shows the first quartile below the median change. *MeDi* Mediterranean diet*, SUVR* standardised uptake value ratio

Graphical representation of the change in SUVR per year between MeDi score tertiles and ‘high’ / ‘low’ fruit intake demonstrates higher overall rates of Aβ accumulation in those participants with ‘low’ MeDi adherence (tertile 1) and with ‘low’ fruit intake (Fig. 1); there were however, still four participants in the ‘high’ MeDi adherence tertile (tertile 3) with relatively high Aβ accumulation rates. Further investigation of these four participants found all were *APOE* ε4 allele carriers.

## Discussion

The aim of the current study was to assess the relationship between MeDi adherence, intake of individual MeDi components, and change in cerebral Aβ burden over 36 months, in cognitively normal healthy control participants of the AIBL Study who were categorised as “Aβ accumulators”, and thus considered to be on the AD pathway. Increasing MeDi adherence was associated with less Aβ accumulation in our cohort (adjusted for confounders), with fruit intake the MeDi component seemingly conferring the greatest benefit. These data suggest that MeDi adherence is beneficial for maintaining brain health in Australian older adults, by slowing accumulation of cerebral AD pathology.

The health benefits of the MeDi are well-established, with a plethora of studies linking adherence to this dietary pattern to low morbidity and mortality for numerous chronic diseases, including diabetes, cardiovascular and cerebrovascular disease (e.g., refs ^1,2,17^.); conditions which are themselves risk factors for AD^3,4,18^. The direct relationship between MeDi adherence and decreased AD risk has also been investigated, with higher MeDi adherence associated with lower incidence of mild cognitive impairment (MCI) and AD^7,19^, as well as with slower cognitive decline among cognitively normal older adults^5^. Furthermore, the results of a recently completed, four year, randomised clinical trial (RCT), revealed that cognitively normal participants adhering to a MeDi, supplemented with either extra-virgin olive oil or mixed nuts, demonstrated cognitive improvement compared to the control group who experienced age-related cognitive decline^20^.

Few studies however, have examined the relationship of MeDi adherence to AD-related neuroimaging outcome measures. Investigations utilising magnetic resonance imaging (MRI) have found higher MeDi adherence to be associated with greater cortical thickness^8^, whilst similarly, lower MeDi adherence has been shown to be predictive of total brain atrophy over three years^9^. A recent cross-sectional study of individuals with MCI or subjective memory impairment (*N* = 44) reported a relationship between greater frequency of following a Mediterranean-type diet and less brain Aβ/tau burden as assessed by 2-(1-(6-[(2-[F-18]fluoroethyl)(methyl)amino]-2-naphthyl)ethylidene)malononitrile (^18^F-FDDNP); a pan-amyloid PET tracer that binds to both Aβ plaques and tau deposits. Unfortunately, however, the lack of selectivity of the tracer makes it impossible to determine whether the diet was associated with Aβ, tau, or a combination of both^21^. To-date, only one study has specifically examined the relationship between MeDi adherence and cerebral Aβ load. Consistent with our findings, the authors reported reduced cerebral Aβ load among individuals with high MeDi adherence^10^. However, this study was cross-sectional in design and utilised a relatively small cohort (*N* = 45) of cognitively normal adults: Factors, the authors themselves described as limitations, which are addressed in the current study, being both longitudinal in design, and utilising data collected from a larger cohort of cognitively normal older adults (*N* = 77).

In our cohort, each one point MeDi score increase was associated with a 0.01 SUVR unit decrease in the accumulation of Aβ over three years; i.e., an individual who increased their MeDi adherence from 0 to 9 could potentially demonstrate a 0.09 SUVR unit decrease in the accumulation of Aβ over three years compared to a 0.01-unit decrease if they increased their MeDi adherence from 0 to 1. Consistent with estimates of brain Aβ accumulation from Villemagne et al.^12^, the average annual increase in brain Aβ in our cohort of known cognitively healthy “Aβ accumulators” was 0.05 SUVR units, which over 36 months would equate to an increase of 0.15. Thus, our findings suggest that adherence to the MeDi could potentially reduce this accumulation of Aβ by up to 60% over three years; thereby presenting a feasible strategy to slow the accumulation of cerebral AD pathology.

How MeDi adherence might exert such beneficial effects remains to be determined. Specifically, it cannot be determined from the current study whether MeDi adherence is associated with reduced Aβ production and deposition, increased Aβ clearance, or a combination of both. Indeed, the MeDi is an abundant source of vitamin A (both preformed vitamin A: retinol and its esterified form, retinyl ester, found in foods from animal sources, including dairy products, fish, and meat; and pro-vitamin A carotenoids, found in plant pigments), which has been shown to demonstrate anti-oligomerization effects on Aβ in vitro^22^. Further, dietary deficiency of vitamin A increases cerebral Aβ deposition in animal models^23^. The monounsaturated omega-9 fatty acid, oleic acid comprises the majority of olive oil, which is consumed readily as part of the MeDi. Oleic acid has been shown to inhibit activity of the amyloidogenic pathway enzyme Beta-secretase 1 (BACE1) in vitro^24^, and to ameliorate amyloidosis in a cellular model of AD^25^. MeDi adherence may also positively modulate levels of Aβ-degrading proteases. For example, MeDi adherence has been associated with reduced levels of fasting insulin ^26^, which consequently increases levels of ‘free’ insulin-degrading enzyme; a well-established Aβ-degrading protease^27^. The MeDi has also been linked to beneficial effects for multiple determinants of cardiovascular disease risk, including decreased low density lipoprotein cholesterol and increased high density lipoprotein (HDL) cholesterol (see ref. ^28^ for an overview of associated RCTs). HDL maintains Aβ solubility within plasma and cerebrospinal fluid^29^, and increasing levels of HDL has been postulated to enhance lipoprotein receptor-related protein clearance of Aβ (e.g ref. ^30^). Indeed, one might hypothesise that the most plausible explanation for the observed association between MeDi adherence and reduced Aβ accumulation in the current study is a complex milieu of beneficial effects rather than a single consummate anti-Aβ mechanism.

Our results suggest that fruit intake is an important element of MeDi adherence which confers benefit in our cohort, specifically; higher intake was associated with less accumulation of Aβ. Whilst the potential mechanism is unclear, we speculate that it may relate to polyphenolic content. For example, the citrus flavonoid nobiletin has been shown to decrease Aβ burden in the brains of AD mouse models^31^, whilst (−)-epicatechin (abundant in multiple fruits) has been associated both with reduced Aβ pathology in a double transgenic mouse model of AD, and potent inhibition of amyloidogenic processing in vitro^32^. The high concentration of vitamin C in fruits consumed as part of the MeDi (e.g. oranges, grapefruit and strawberries) may also contribute to slowing accumulation of cerebral AD pathology. Vitamin C supplementation has been shown to reduce amyloid plaque burden in the brain of a transgenic mouse model of AD which had been genetically engineered to be unable to synthesize its own vitamin C (important as the ability of rodents to synthesize vitamin C with no reliance on dietary supply has hampered previous investigations)^33^. Moreover, vitamin C has been shown to inhibit amyloid fibril formation in vitro^34^.

There are limitations to our report that require consideration; this is an observational study, therefore we can draw no conclusions regarding causality. Further, the CCVFFQ utilised in this study relies on participants’ estimations of food intake over the previous year; this is a common limitation of studies of diet and can potentially lead to misclassification of dietary intake due to limited accuracy. This is particularly important with regards to cognitively impaired participants, thus, to circumvent this limitation, only cognitively normal older adults were included in the current analysis. Nevertheless, we recognise that ideally, a targeted intervention substantiated with biomarker measures for actual consumption/uptake would be utilised to both determine causality and to evade the limitations of self-report.

Many aspects of our study, however, provide confidence in our findings. We have utilised a well-characterised cohort thereby increasing the internal validity of our results. We have taken a rigorous approach to statistical analysis. The dietary data were collected using an instrument previously validated in epidemiological studies^13,14^. Moreover, our findings are consistent with many published studies which describe the benefits of MeDi adherence in the context of both AD risk and severity of cerebral pathology (e.g., refs ^6,7,9,10,17^).

This is the first study to assess the relationship between MeDi adherence and change in cerebral Aβ burden over time in older adults. In summary, the results of this study highlight the potentially beneficial impact of MeDi adherence on slowing rates of cerebral Aβ accumulation in cognitively unimpaired older adults. Whilst validation of our findings is required using a targeted intervention, when our results are considered collectively with previous data linking the MeDi to improved cognition, it appears that MeDi adherence warrants further investigation in the quest to delay or ideally prevent AD onset.
